# Beyond the Bench: Online and On Track with Veggie-Mon

**Published:** 2005-11

**Authors:** Tanya Tillett

Too much computer time may not be good for kids, but sometimes surfing the Internet can be a wholesome activity, especially when it involves websites that help children learn how to make informed choices about their own health. One such site is the Veggie-Mon website at http://www.veggie-mon.org/. Created in 2000 by the Community Outreach and Education Program (COEP) of The Center for Research on Environmental Disease, a joint NIEHS center of The University of Texas M.D. Anderson Cancer Center and The University of Texas at Austin, the Veggie-Mon website has informed thousands of kids about the choices they can make to lead a healthy life.

The Veggie-Mon site introduces concepts of environmental risk factors and disease prevention to elementary- and middle-school students in a compelling and comprehensible way. “The goal of the site is to inform students, even young ones, that they can have an important and long-term impact on their own health by reducing their exposure to environmental risk factors and improving their diet,” says COEP director Robin Fuchs-Young.

The homepage offers three portals, one for students in grades 4–6, one for students in grades 7–8, and one for teachers. Both student portals present information on three main topics: nutrition, sun and ultraviolet (UV) exposure, and tobacco use. According to Fuchs-Young, these are among the most important environmental risks faced by school-age children, and are also some of the risks that are most easily mitigated.

Each visitor is accompanied through the different sections by Veggie-Mon himself, a character reminiscent of a walking, talking artichoke who offers site navigation tips and provides extra details on the information presented. Each of the three sections has information that is both informative and fun. Along the way, Veggie-Mon encounters different acquaintances who help him explain the subject matter.

In the Nutrition section, students meet Strawberry Girl, an advocate of healthy eating habits. Here students can learn how to make healthy food choices through an illustrated food pyramid, and can also find recipes for delicious, wholesome snacks like a strawberry banana blast or a peanut butter and honey sandwich.

The Sun and UV section features Sunspot, a character who discusses some of the dangers of too much sunlight. In this section, students learn how fish research is helping scientists study the connection between sun exposure and skin cancer, and they can also take Sunspot’s quiz to gauge how much they’ve learned.

In the Tobacco Road section, students meet Igna-Ray-Mouse, a misinformed rodent who has decided to smoke. Here they can take a virtual journey down Tobacco Road with Igna-Ray-Mouse and learn how advertising messages and peer pressure may be used to try to convince them to smoke. At each fork in the road, evidence is presented to prove that choosing to smoke is a bad idea.

Other tools on the site include a submission form to send questions to real scientists, a glossary, and a “laboratory” with instructions for simple experiments that students can conduct themselves. Each section also includes age-appropriate games and puzzles.

Teachers have their own features on the site. In a password-protected area, they can access lesson plans and provide feedback on how the website has helped them with classroom activities. Educators also contribute directly to the development of the website. During a 4- to 6-week educator fellowship held each summer at The Center for Research on Environmental Disease, teachers from grades K–12 help the COEP staff translate center research findings into age-appropriate content.

The COEP regularly revises the Veggie-Mon website to improve its usefulness for both students and teachers. Next up for the site is an exercise unit for the Nutrition section that will offer suggestions for fun and safe activities as well as information on healthy weight maintenance.

## Figures and Tables

**Figure:**
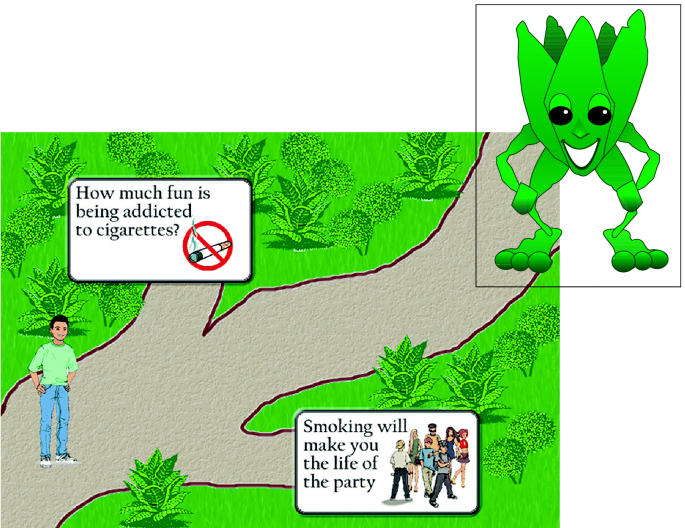
A virtual journey to real health. The Veggie-Mon website uses a cartoon character (inset) to introduce students to concepts of good diet, nutrition, and healthy lifestyle choices. In one activity, students take a virtual journey along Tobacco Road and read billboards with messages about smoking.

